# Radical-Mediated Enzymatic Polymerizations

**DOI:** 10.3390/ijms17020195

**Published:** 2016-02-02

**Authors:** Scott R. Zavada, Tsatsral Battsengel, Timothy F. Scott

**Affiliations:** 1 Macromolecular Science and Engineering Program, University of Michigan, Ann Arbor, MI 48109, USA; szavada@umich.edu; 2 Department of Chemical Engineering, University of Michigan, Ann Arbor, MI 48109, USA; tsatsa@umich.edu; 3 Department of Chemical Engineering and Macromolecular Science and Engineering Program, University of Michigan, Ann Arbor, MI 48109, USA

**Keywords:** polymerization, enzymes, oxidase, peroxidase, vinyl, thiol–ene, ATRP, oxidative coupling

## Abstract

Polymerization reactions are commonly effected by exposing monomer formulations to some initiation stimulus such as elevated temperature, light, or a chemical reactant. Increasingly, these polymerization reactions are mediated by enzymes―catalytic proteins―owing to their reaction efficiency under mild conditions as well as their environmental friendliness. The utilization of enzymes, particularly oxidases and peroxidases, for generating radicals via reduction-oxidation mechanisms is especially common for initiating radical-mediated polymerization reactions, including vinyl chain-growth polymerization, atom transfer radical polymerization, thiol–ene step-growth polymerization, and polymerization via oxidative coupling. While enzyme-mediated polymerization is useful for the production of materials intended for subsequent use, it is especially well-suited for *in situ* polymerizations, where the polymer is formed in the place where it will be utilized. Such polymerizations are especially useful for biomedical adhesives and for sensing applications.

## 1. Introduction

Synthetically-generated polymers are used in countless applications, from industrial paints, coatings, and sealants, to plastics utilized in automotive and aerospace industries, to biomedical materials such as surgical glues or dental fillings. Many methods for generating polymers exist, some of which are performed in a reactor for subsequent use, while others are better-suited for the formation of polymers *in situ*—that is, the location where the material will the utilized. There are often restrictions on the types of materials used in making polymers or in the environments where the polymers are made. For example, biomedical adhesives utilized *in vivo* need to be biocompatible and thus cannot contain materials known to cause adverse reactions within the body. One approach being investigated as a means to overcome these limitations is the utilization of enzyme-mediated reactions. As will be discussed throughout this review, enzymes have been extensively employed to initiate or otherwise influence polymerization reactions.

Enzymes are proteins that catalyze biochemical reactions. By binding a substrate to the active site of an enzyme, the activation energy of the reaction can be decreased, resulting in a substantial reaction rate increase. For example, the enzyme orotidine 5’-phosphate decarboxylase accelerates the orotidine 5’-monophosphate decarboxylation rate by 10^17^ times the uncatalyzed reaction rate [1]. Whereas enzymes are responsible for all naturally synthesized biomacromolecules, they are also capable of retaining catalytic properties outside of biological systems and thus have been employed for *in vitro* polymer synthesis*.* This utilization of enzymes is becoming increasingly attractive owing to the progressive cost reductions that accompany advances in biotechnology.

The first enzymatic polymerization was reported in 1951 by Parravano who demonstrated an oxidase-mediated polymerization of methyl methacrylate [2]. Since then, many enzymes have been investigated as a means to initiate or control polymerization reactions in order to take advantage of their strengths, including high catalytic turnover, environmentally-friendliness (e.g., production from renewable resources), facile control of polymer architecture, and easy separation from products. Many, though certainly not all [3,4,5,6,7,8,9], enzyme-mediated polymerization reactions involve free radicals; these free radical species are generated either directly by the enzyme or via a secondary reaction involving an enzyme-derived product, methods that eliminate the requirement for the thermal energy or irradiation used to initiate conventional free radical polymerization. These enzymatic, radical-generating reactions all utilize reduction and oxidation steps and, accordingly, most of the enzymes used in these processes are termed oxidoreductases. Commonly, these enzymes utilize either oxygen or hydrogen peroxide as a substrate and are generally known as oxidases or peroxidases, respectively. The most commonly utilized enzymes for radical-mediated polymerization reactions include horseradish peroxidase, glucose oxidase, and laccase. Through different reactions mechanisms, these three enzymes, as well as several others with similar behavior, are able to initiate, catalyze, or otherwise influence polymerization reactions.

## 2. Enzyme-Mediated Polymerization Reactions

### 2.1. Chain-Growth Free Radical Polymerization

**Figure 1:**
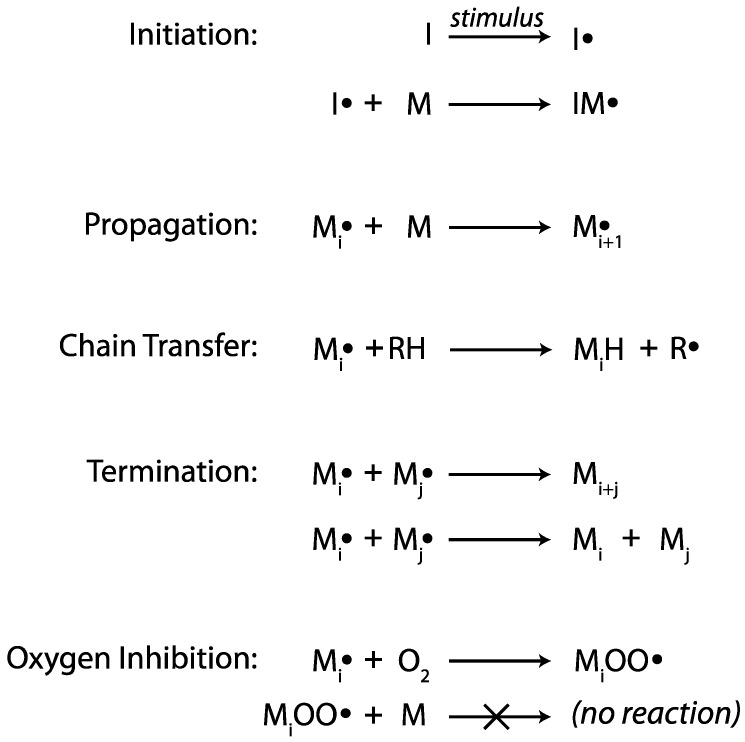
Chain-growth polymerization reactions [10,11,12]. I = an initiator, M = vinyl monomer, M_i_ or M_j_ = polymer with, *i* or *j* repeat units, respectively, O = oxygen, H = hydrogen, RH = a compound with a readily abstractable hydrogen, R = RH after hydrogen abstraction.

The chain-growth free radical polymerization of vinyl monomers (e.g., acrylates, methacrylates, acrylamides, and styrenics) is utilized in a broad array of applications, including plastics, coatings, adhesives, and sealants. Polymerization commences upon an initiation step where a radical, generated by exposing a thermal, photo, or redox initiator to its associated stimulus (heat, light, or chemical, respectively), adds across the carbon-carbon double bond of a vinyl monomer, generating a carbon-centered radical [10,11]. This radical, in turn, reacts with another vinyl monomer, increasing the size of the molecule and again regenerating a carbon-centered radical; these propagation reactions rapidly repeat and the polymer chain grows quickly. Polymerization ceases through one of several termination reactions, including combination and disproportionation, that eliminate the radical, or through chain transfer, a side reaction where the radical on the growing chain abstracts a labile hydrogen, stopping propagation on the original chain and leading to the initiation of a second growing chain. Another common side reaction occurs when an active radical on the growing chain propagates to molecular oxygen to yield a peroxy radical (see Figure 1). Unlike carbon-centered radicals, the peroxy radical reacts sluggishly with monomers, drastically reducing the reaction rates, and causing polymerization to nearly cease [12]. Consequently, the radical-mediated chain-growth polymerization of vinyl monomers is typically very susceptible to oxygen inhibition.

There are numerous approaches to perform these radical-mediated polymerization reactions. Bulk polymerization involves only monomer and initiator, while solution polymerization has the monomers and initiators dissolved in a solvent that also keeps the generated polymer in solution [13,14]. In contrast, precipitation polymerization utilizes a solvent where the generated polymer is insoluble, and will thus precipitate out of solution once it reaches a sufficiently high molecular weight [15]. Instead of dissolving the monomers and initiators in a solvent, suspension polymerization has them dispersed in a non-solvent continuous phase, and subsequent polymerization converts the liquid droplets into polymer beads [16]. If instead the initiator is soluble in the continuous phase, often water, the resulting emulsion polymerization process can be used to generate an aqueous polymer latex [17,18]. In all of these methods, linear or branched polymers can be generated from monofunctional reactants, with cross-linked polymer networks readily attained through the utilization of multifunctional monomers. For many industrial and commercial applications, *in situ* bulk polymerization is especially common and is used to generate numerous types of coatings and adhesives. Other common applications, especially for biomedical use, involve the *in situ* generation of hydrogels by cross-linking an aqueous solution of multi-functional monomers.

As an alternative to thermal-, photo-, and organometallic redox-initiators, enzymes have been used to generate the radicals necessary for the initiation of chain-growth polymerization. An early report by Derango and coworkers demonstrated that several enzymes (horseradish peroxidase, xanthine oxidase, chloroperoxidase, and alcohol oxidase) would, in the presence of suitable substrates, polymerize aqueous solutions of acrylamide and hydroxyethylmethacrylate (HEMA) [19]; as the polymerization proceeded, the polymer would either precipitate out of solution or would form a hydrogel. The enzymes used by the Derango group are all oxidoreductases―enzymes that concurrently reduce one substrate while oxidizing another. Commonly employed oxidoreductases include peroxidases, which reduce hydrogen peroxide, and oxidases, which reduce molecular oxygen; the substrate that is being oxidized depends upon the particular enzyme, but may be one of the monomers themselves or an additional compound known as a mediator. Three oxidoreductase enzymes, horseradish peroxidase, glucose oxidase, and laccase, dominate the discussion here.

#### 2.1.1. Horseradish Peroxidase

**Figure 2:**
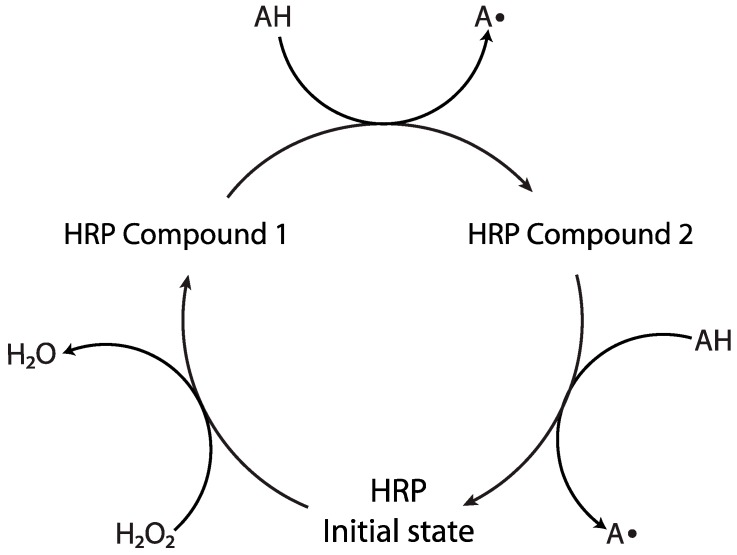
Horseradish peroxidase (HRP) catalyzes the reduction of hydrogen peroxide to water by oxidizing two equivalents of a hydrogen-donating mediator AH. HRP compounds 1 and 2 are oxidized forms of HRP that are each capable of oxidizing the mediator AH, yielding radicals that may be utilized for the initiation of radical-mediated polymerization [21,22,23]. Arrows show reaction direction.

One of the most commonly used enzymes for polymerization reactions is horseradish peroxidase (HRP), a heme-containing enzyme with a molar mass of approximately 40 kDa [20] that is capable of generating radicals by reducing hydrogen peroxide to water while oxidizing two equivalents of a hydrogen-donating mediator (see Figure 2) [21,22,23]; the oxidation of the mediator affords radicals capable of initiating polymerization. HRP, derived from the horseradish root, contains several different isoenzymes of which the C isoenzyme (HRP C) is the most commonly used [24]. HRP has been utilized in many applications, including waste treatment, where it has been utilized in the removal of phenolic compounds from wastewater [25,26,27], and immunoassay-type biochemical testing [28,29,30].

**Figure 3:**
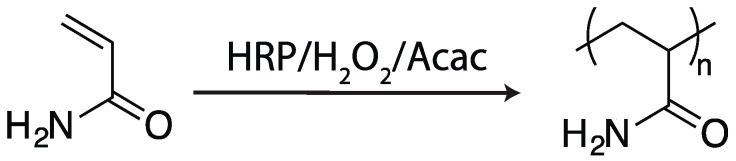
Acrylamide is polymerized into poly(acrylamide) in the presence of HRP, hydrogen peroxide, and acetylacetone (Acac) [22,31,32,33].

Amongst its many roles in catalyzing polymerization reactions, HRP has been utilized extensively for the solution polymerization of vinyl monomers. Expanding on the early work described by Derango and coworkers [19], Lalot and coworkers demonstrated that acrylamide is readily polymerized into poly(acrylamide) by utilizing HRP, hydrogen peroxide, and a diketone mediator, commonly acetylacetone, as a ternary initiating system (see Figure 3); the reaction proceeds at room temperature upon combining the aqueous solution of acrylamide with all three components of the initiating system [22,31,32,33]. A recurring theme of enzymatic polymerization is that, for many systems, no reaction proceeds until all necessary components are present, opening up the possibility that such initiating systems may be suitable for *in situ* polymerization. For the system described by Lalot and coworkers, if only two of the three initiating components were added, no reaction proceeded until the addition of the third [31]. In the presence of all three (utilizing 1.8 g/L HRP, 0.01 M H_2_O_2_, and 0.017 M acetylacetone), polymerization proceeded over several hours, leading to the formation of atatic poly(acrylamide) with a number-average molecular weight (M_n_) of between 150 and 460 kg/mol and reaction conversions of 70%–90%. One significant reason for this extended reaction period was an approximately one hour induction period where no polymerization occurred, likely attributable to oxygen-induced inhibition. Notably, a decrease in reaction yield, from 92% to 72%, was observed as the acrylamide concentration was raised from 1 to 5 M; although the authors attributed this reduced yield to an increase in viscosity, enzyme deactivation via denaturation would yield similar results.

It is interesting to note that, whereas Lalot reported no polymerization in the absence of acetylacetone [31], Derango and coworkers reported the utilization of HRP without the addition of any mediator [19]. This apparent discrepancy is readily explained in that the Derango group used significantly more hydrogen peroxide; thus, the addition of a mediator permits substantially lower hydrogen peroxide concentrations. This is tremendously useful as hydrogen peroxide can deactivate HRP, a problem that is particularly severe at high hydrogen peroxide concentrations [22]. As a demonstration, in the results provided by Lalot, no polymerization proceeded in the absence of the mediator and would, as noted above, start immediately upon its addition [31]; however, delaying the addition of the mediator permitted the hydrogen peroxide to degrade HRP, leading to a significant decrease in yield. For example, in the polymerization of an aqueous solution of acrylamide monomer, when all three components (e.g., HRP, acetylacetone, and hydrogen peroxide) were present together, the reaction yield was 87%. Similar results were obtained when either HRP or acetylacetone were initially omitted from the formulation and subsequently added one hour later. In contrast, if the acetylacetone was initially omitted and then added later, no polymerization was observed; thus, the hydrogen peroxide necessary for initiation also participated in a deactivation reaction that irreversibly yielded an inactive, non-catalytic form of HRP.

An in-depth exploration of the polymerization of acrylamide by HRP, hydrogen peroxide, and acetylacetone was performed by Wen and coworkers to provide insight into some of the questions raised by earlier work [34]. Regarding the lengthy inhibition periods observed by Lalot [31], Wen found that the inhibition period prior to polymerization was caused by oxygen inhibition. This inhibition time could be reduced or eliminated by increasing the concentration of acetylacetone, to as high as 0.13 M, as this led to an increase in the radical generation rate; as these radicals reacted with and consumed oxygen, propagation reactions successfully competed with the inhibition reactions allowing the polymerization to proceed once the oxygen concentration was sufficiently low. Owing to the reduced inhibitory period, reactions times were significantly shorter than those observed by Lalot; however, the synthesized poly(acrylamide) batches were similar to those described by Lalot, having M_n_s of 200 to 630 kg/mol and dispersities (*Đ*), a measure of the molecular weight distribution width, of 2.0 to 3.0, with yields of 72% to 97% [34].

In addition to generating soluble polymers in solution, HRP/hydrogen peroxide/acetylacetone ternary initiating systems have been used to generate cross-linked hydrogels by utilizing aqueous solutions of multi-functional vinyl monomers. In work by Wang and coworkers, this method was used to copolymerize acrylated human serum albumin and *N*,*N*-dimethylacrylamide; upon addition of the initiating system component, gels formed within 1 min [35]. These experiments again confirmed the inhibitory influence of hydrogen peroxide, as no gelation was observed when it was used in excess. Interestingly, the residual HRP, effectively immobilized after gelation, retained much of its activity in the hydrogel matrix.

HRP-mediated polymerization has also been utilized to generate polymers on surfaces [36]. Here, poly(acrylamide)-grafted particles were formed by polymerizing acrylamide in the presence of HRP, hydrogen peroxide, and β-diketone-functionalized silica particles. As polymerization was initiated at the β-diketone moiety located on the surface of the silica particles, this process resulted in a core-shell morphology, yielding poly(acrylamide) layers with thicknesses of 15–192 nm; the poly(acrylamide) itself had M_n_ ranging from 63 to 273 kg/mol, with *Đ* values from 1.5 to 3.0. Once again, both the initiating and inhibitory roles of hydrogen peroxide were confirmed in these experiments as no poly(acrylamide) shell was formed either in its absence or its presence in excess.

Whereas enzyme-mediated polymerization is naturally suited for use with aqueous monomers, given that many enzymes naturally prefer an aqueous and pH-controlled milieu, HRP is sufficiently tolerant of some organic solvents that it may be utilized with monomers that require the addition of non-trivial amounts of solvent for dissolution. For example, methyl methacrylate, a monomer commonly polymerized via a free-radical mechanism, has poor solubility in water but does dissolve in aqueous solutions of either tetrahydrofuran (THF) or dioxane. By again utilizing the HRP, hydrogen peroxide, and acetylacetone ternary initiating system in a 25% THF aqueous solution, poly(methyl methacrylate) (PMMA) was readily generated with a M_n_ of 72 kg/mol and a *Đ* of 3.1 at 85% yield, and was >80% syndiotactic [37]. In an example that used even more hydrophobic monomers, styrene, 4-methylstyrene, and 2-vinylnaphthalene were all readily polymerized by HRP when dissolved in a suitable solvent system and in the presence of hydrogen peroxide and a mediator [21]. The authors of this study investigated solvent and mediator effects and found that both significantly influenced yield and molecular weight. Dimethylformamide (DMF), methanol, or dioxane as solvent resulted in particularly low yields, while the best solvent system was found to be THF:H_2_O in a 4:1 volumetric ratio. When styrene was dissolved in this solvent system and polymerized in the presence of acetylacetone, the M_n_ was nearly 32 kg/mol with a yield of 21.2%, while replacing acetylacetone with cyclopentadiene increased the M_n_ and yield to 68 kg/mol and 59.4%, respectively, affording a poly(styrene) that was nearly completely atactic. Interestingly, the authors reported that the mediator was incorporated into the generated polymer, introducing the potential for post-polymerization modification. Interestingly, the authors reported <5% yields during control experiments where iron(II) salts were used in place of HRP, although hydroxyl radicals, generated as a result of the Fenton reaction [38,39,40], would be anticipated to effect polymerization. This is perhaps unexpected as the utilization of the non-enzymatic Fenton reaction in combination with the enzymatic production of hydrogen peroxide has been previously reported [41]. The lack of iron-mediated polymerization here was likely owing to high (e.g., 0.16 M) Fe^2+^ concentrations leading to polymerization-suppressing inhibitory and terminating reactions [42].

As an alternative to solution polymerization in non-aqueous systems, HRP-mediated emulsion polymerization has also been utilized with monomers having limited water solubility. To illustrate, an emulsion of styrene in water, stabilized by the surfactant sodium dodecyl sulfate (SDS), was polymerized under anaerobic conditions by the familiar HRP, hydrogen peroxide, and acetylacetone ternary initiating system [43]. As the components of the initiating system are all water soluble, polymerization proceeded through an emulsion polymerization mechanism, where polymers growing within surfactant-stabilized micelles yielded stable particles. This resulted in the formation of stable poly(styrene) particles, with diameters of 30–50 nm and yields typically between 40% and 60%; the polymer had M_n_ ranging from 173 to 516 kg/mol with *Đ* of between 3 and 8, with both being influenced by hydrogen peroxide concentration. Another approach to enzyme-mediated emulsion polymerization was demonstrated by utilizing HRP immobilized on a silicon wafer [44]. The immobilization of HRP to a solid substrate conceivably simplifies its removal from the reaction mixture and allows its reuse. Emulsions of ethylene glycol dimethacrylate, stabilized by cetyltrimethylammonium bromide, were polymerized with the immobilized HRP, hydrogen peroxide, and acetylacetone. Although conversions were low―less than 5%―control experiments using free HRP yielded similar results. While the polymerization results described here were somewhat disappointing, given the very low yields, the procedure did successfully demonstrate the reusability of immobilized HRP.

#### 2.1.2. Glucose Oxidase

**Figure 4:**
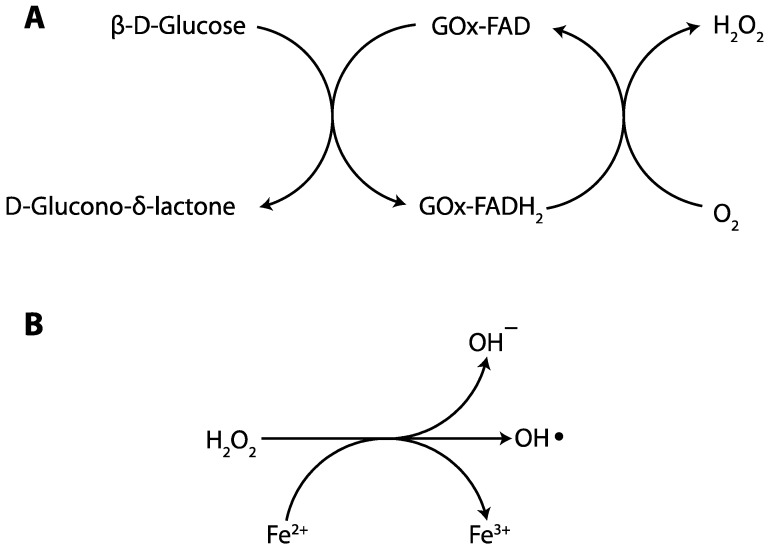
(**A**) Glucose oxidase catalyzes the reduction of oxygen and oxidation of glucose into hydrogen peroxide and gluconolactone, respectively [49]; (**B**) Hydrogen peroxide is readily converted to hydroxyl radicals via the Fenton Reaction with Fe^2+^ ions [38,39,40]. Arrows show reaction direction.

**Figure 5:**
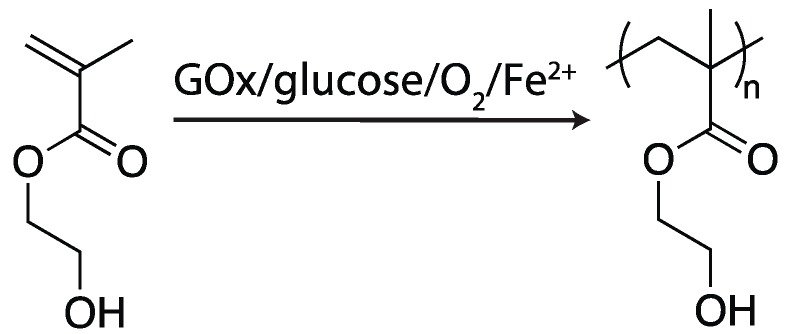
2-Hydroxyethyl methacrylate (HEMA) is polymerized into poly(HEMA) in the presence of glucose oxidase (GOx), glucose, oxygen, and Fe^2+^ [41].

Another oxidoreductase, glucose oxidase (GOx), has also been utilized to initiate vinyl polymerization. GOx is a dimeric glycoprotein obtained from various sources and has been extracted from several fungi including Aspergillus [45,46] and Penicillium [46,47]. This enzyme is composed of two identical polypeptide chain subunits connected by a disulfide bond [46,47], with a total molar mass that varies from 130 to 175 kDa [46], and relies on a tightly bound cofactor, flavin adenine dinucleotide (FAD), for its catalytic activity. GOx utilizes molecular oxygen as an electron acceptor to catalyze the oxidation of β-d-glucose to d-glucono-δ-lactone and hydrogen peroxide [45,48]. This reaction initially proceeds by the enzymatic oxidation of glucose to d-glucono-δ-lactone, which is then non-enzymatically hydrolyzed to gluconic acid and is generally of little importance to subsequent reactions, while the cofactor FAD is reduced to FADH_2_. Subsequently, the reduced cofactor is reoxidized by molecular oxygen to yield hydrogen peroxide (see Figure 4a) [49]. The *in situ* generated hydrogen peroxide can then be converted into initiating radicals through several strategies. An early example, by Iwata and coworkers [41] utilized Fenton chemistry (see Figure 4b) [38,39,40] whereby the oxidization of Fe^2+^ to Fe^3+^ concomitantly reduced hydrogen peroxide to a hydroxyl anion and a hydroxyl radical – the polymerization initiating species. Here, aqueous solutions of 2-hydroxyethyl methacrylate (HEMA), dissolved in a 0.1 M acetate buffer solution, were polymerized by the addition of GOx, glucose, and ammonium ferrous sulfate ((NH_4_)_2_Fe(SO_4_)_2_) (see Figure 5). The authors confirmed that no polymerization proceeded in the absence of oxygen or Fe^2+^, but formulations lacking either of these components would begin to polymerize once the missing components was added. This concept was explored in depth in a series of papers by Bowman and coworkers where hydrogels were formed from aqueous solutions of acrylate monomers [42,50,51,52,53]. In their earliest paper [42], hydrogels were formed from poly(ethylene glycol) diacrylate (PEGDA) and 2-hydroxyethyl acrylate within minutes of adding the initiating system components. The oxygen required for the reaction as supplied simply by the dissolved gases normally present (about 10^−3^ M) in the monomers. While oxygen is known to inhibit the chain-growth polymerization of acrylates, the GOx-mediated reaction consumed the oxygen sufficiently quickly to enable polymerization to proceed without significant oxygen inhibition. Regarding control over reaction rates, both Fe^2+^ and glucose played significant roles. Increasing the glucose concentration increased the polymerization rate until a point is reached where the system is saturated; as GOx is limited in how fast it can process glucose, excess glucose (greater than 1.0 × 10^−3^ M glucose with 6.25 × 10^−7^ GOx) did not increase reaction rates further once this point was reached. In contrast, whereas polymerization rates increase with raised Fe^2+^ at low Fe^2+^ concentrations, rates began to decrease at high Fe^2+^ concentrations (approximately 3 × 10^−4^ M). This iron-mediated inhibition was attributable to radical-consuming side reactions involving either Fe^2+^ or Fe^3+^ ions. Despite these minor limitations, this initiating system was used to generate cell-encapsulating hydrogels, where the high resultant cell viability demonstrated the cytocompatibility of the initiating system components, including the residual GOx [42]. Thus, the hydrogels generated by this method could be suitable for utilization in biomedical applications.

As noted above, a peculiarity of HRP-mediated polymerization is the rapid enzyme degradation by hydrogen peroxide [21,22]. This represents a serious challenge as hydrogen peroxide is the most common oxidant in the redox reaction catalyzed by HRP. While it is possible to circumvent HRP deactivation by simply adding the hydrogen peroxide slowly, limiting the concentration present at any given time, this manual and tedious solution is not always applicable, notably during *in situ* polymerizations. Instead of manually controlling the concentration, hydrogen peroxide can be generated *in situ* by utilizing GOx, an approach that minimizes the hydrogen peroxide concentration present at any time as the GOx-generated hydrogen peroxide is quickly consumed by HRP. Such a bienzymatic system has been utilized both for thiol–ene reactions and for the oxidative coupling of phenols (both of which are discussed at length below), and has also been used for vinyl polymerization. For example, the GOx/HRP system was utilized to form bioinorganic hybrid hydrogels by polymerizing poly(ethylene glycol) methacrylate (PEGMA) in the presence of calcium niobate (CNO) nanosheets [54]. Self-assembled sandwich structures, generated by combining HRP and CNO, were dispersed in an aqueous solution and polymerization would proceed after PEGMA, GOx, and glucose were added in the presence of molecular oxygen. During polymerization, the assembled HRP/CNO structures would exfoliate, leading to the formation of a composite structure. As the enzymes were generally left unaffected by the polymerization, they were still able to function as catalysts even after hydrogel formation. To evaluate the reusability of the immobilized enzymes, pyrogallol and glucose were added to the hydrogels; an enzymatic cascade, similar to the polymerization reaction, would proceed and the hydrogen peroxide, generated by GOx, would induce HRP-mediated oxidation of pyrogallol into purpurogallin. Even after five cycles, the enzymes retained over 80% of their activity. Furthermore, the authors also found that the presence of the CNO nanosheets aided in preventing enzyme thermal deactivation, permitting these materials to be used under a broader range of conditions and suggesting their utility in a variety of sensor and purification applications.

Bienzymatic systems have also been employed to solve other problems, notably circumventing oxygen inhibition. As discussed above, the radical-mediated polymerization of vinyl monomers is particularly susceptible to oxygen inhibition. The presence of GOx in a bienzymatic initiating system thus functions not only in its aforementioned role for the *in situ* production of hydrogen peroxide but also as an oxygen scavenger, deceasing the oxygen concentration such that propagation is no longer inhibited. In one example, the enzyme-mediated polymerization of 3-aminopropyl methacrylamide (APMA) was utilized to effect gold nanoparticle aggregation; once a sufficient degree of polymerization was reached, the resultant aggregation of the gold nanoparticles afforded a color shift readily monitored by changes in the visible spectrum [55]. In this system, both GOx and HRP were utilized and it was the presence of GOx that permitted the polymerization reaction to proceed without inhibition under atmospheric conditions.

Not all GOx-mediated reactions utilize molecular oxygen as the oxidant. Polymerization of PEGDA has been initiated by GOx (4 × 10^−6^ M), in the presence of glucose, catalyzing the reduction of *N*-hydroxy-5-norbornene-2,3-dicarboximide (HNDC) into a carbon-centered radical species [56]. Moreover, this reaction still proceeded in the presence of HNDC-conjugated heparin, resulting in a hybrid PEGDA/HNDC-conjugated heparin hydrogel. Unlike the examples of GOx-mediated polymerization described above, molecular oxygen seemingly played no initiating role—all materials were thoroughly degassed prior to use. Such heparin-based hydrogels may be suited for drug delivery as the heparin-degrading enzyme heparanase is frequently overexpressed by some forms of cancer [57].

#### 2.1.3. Laccase

**Figure 6:**
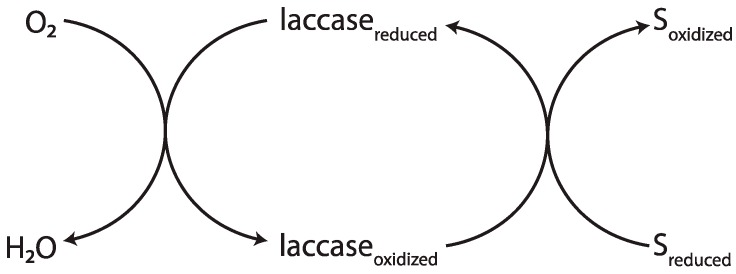
Laccase catalyzes the reduction of oxygen to water by oxidizing an appropriate substrate [59]. Arrows shown reaction direction.

**Figure 7:**
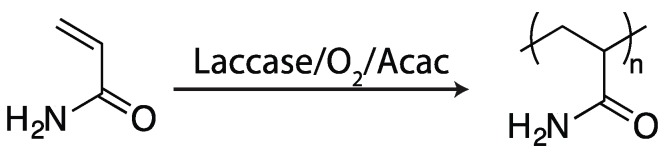
Acrylamide is polymerized into poly(acrylamide) in the presence of laccase, oxygen, and acetylacetone (Acac) [58].

Laccases, copper-containing oxidoreductases that are obtained from a variety of organisms, commonly fungi, including *Pycnoporus coccineus* [58], *Myceliophthora thermophile* [59], *Trametes trogii* [60], and *Trametes versicolor* [61,62,63], catalyze the reduction of molecular oxygen while concurrently oxidizing a hydrogen-donating substrate (see Figure 6) [59]. These reactions result in the formation of radical-bearing species and have been utilized in pulp/paper [64], food/beverage, and waste treatment applications [65,66]. Laccase is of growing interest for polymerization reactions as it is able to directly generate radicals by oxidation of a mediator, commonly acetylacetone, unlike GOx which usually requires a second reaction to afford initiating radicals from the generated hydrogen peroxide. By utilizing a ternary initiating system similar to ones with HRP discussed above, Kobayashi and coworkers demonstrated the polymerization of acrylamide in the presence of oxygen, 0.016 M acetylacetone, and 7.8 g/L laccase (see Figure 7), affording polymer with a M_n_ of 23 kg/mol and a *Đ* of 2.0 at 97% yield [58]. The presence of acetylacetone was necessary to perform the polymerization at room temperature, which otherwise required elevated temperatures to generate polymer. In a study expanding upon this work, Hollmann and coworkers confirmed the lack of polymer formation in the absence of any component of the ternary initiating system (*i.e.,* oxygen, acetylacetone, or laccase) when performing the reaction at room temperature, as well the inhibitory role adopted by oxygen―a problem they termed the “oxygen dilemma” [59]. Furthermore, they explored the limitations of laccase-mediated vinyl polymerization, notably the low activity of the enzyme and its poor stability. As a result of these issues, laccase is far less common than HRP to effect radical-mediated polymerizations; nevertheless, vinyl polymerizations mediated by laccase continue to be explored. To address the dual initiating/inhibiting roles oxygen plays, del Monte and coworkers utilized GOx as an oxygen scavenger [67]. They found that a quaternary initiating system composed of laccase, a mediator, GOx, and glucose would readily induce the polymerization of PEGDA monomers in aqueous solution at 37 °C, leading to the formation of a cross-linked hydrogel. Interestingly, oxygen-mediated inhibitory reactions completely prevented polymerization the absence of GOx and glucose, perhaps surprising as both laccase and GOx utilize oxygen as oxidant and thus each should be capable of removing oxygen from the solution. Also of note is that the hydrogen peroxide generated by GOx did not play any obvious role in the reaction mechanism, as the addition of catalase (an enzyme that removes hydrogen peroxide, converting it into oxygen and water) had minimal influence on the reaction conversion. Notably, instead of using a more typical diketone (e.g., acetylacetone), polyethylene glycol-polypropylene glycol-polyethylene glycol (PEG-PPG-PEG) block copolymers were utilized as macro-mediators; as initiation proceeded from a PEG-PPG-PEG-centered radical, the mediators were incorporated into the polymer network. While the utilization of macro-mediators led to lower conversions than when acetylacetone was utilized, the temperatures required were still sufficiently low to prevent enzyme deactivation. Moreover, the laccase encapsulated within the hydrogel retained 90% of its activity after polymerization, rendering it suitable for pollutant degradation applications.

In an exploration of the pollutant removal ability of a laccase-bearing polymer matrix, Zhang and coworkers first grafted poly(acrylamide) to chitosan in a laccase-mediated polymerization [62]; once again, the necessity of a mediator, acetylacetone, was confirmed as no polymer formed in its absence. The resulting combination of the chitosan-poly(acrylamide) graft copolymer, laccase, and acetylacetone were then successfully used to decolorize a model organic pollutant, malachite green, confirming that laccase was not deactivated during the formation of the chitosan-poly(acrylamide) graft copolymer.

In one particularly sophisticated application, laccase was used in the fabrication of composite nano-gel particles, generated from *N*,*N*’-bis(acryloyl)cystamine and loaded with two enzymes, catalase and superoxide dismutase, for utilization in ultrasound imaging [68]. The formulation contained clusters made from supermagnetic iron oxide and an acrylate- and biotin-functionalized chitosan, as well as the laccase and *N*,*N*′-bis(acryloyl)cystamine needed to form a nano-gel layer around the clusters, and polymerization at 25 °C resulted in the formation of 13 nm diameter particles.

While the laccase examples described thus far have utilized oxygen as a necessary component for initiation, results reported by di Lena and coworkers described the laccase-mediated polymerization of methacrylates via an anaerobic mechanism [69]. They found that an aqueous solution of poly(ethylene glycol) methacrylate could be anaerobically polymerized in the presence of laccase, ascorbic acid, and ethyl 2-bromoisobutyrate; all three components of this ternary initiating system were required to achieve significant reaction conversion, as no reaction occurred in the absence of laccase or ascorbic acid, while reaction conversions of only 2% were attained in the absence of ethyl 2-bromoisobutyrate. This initiating system is notable as it resembles those utilized for atom transfer radical polymerization (ATRP), a living polymerization method used to generate polymers with narrow dispersities (*i.e.,* the breadth of a polymer’s molecular weight distribution) [70]. Unfortunately, despite the superficial similarity to ATRP, the laccase/ascorbic acid/bromo-compound ternary initiating system led to the formation of polymers with a *Đ* of greater than 1.94, much higher than those readily attainable through ATRP (often less than 1.1). Moreover, there was no correlation between molecular weight and reaction conversion—another trait of living radical polymerization. Even though the dispersities were disappointingly high, they found that it was possible to narrow the molecular weight distribution, achieving a *Đ* of 1.35, by utilizing a different living polymerization method, reversible addition fragmentation chain transfer (RAFT) [70]. In subsequent research, by this group and others, there have been many additional reports on enzyme-mediated living radical polymerization.

### 2.2. Living Radical Polymerization

**Figure 8:**
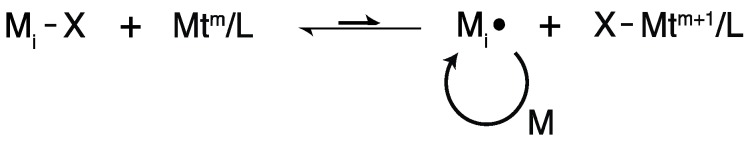
Atom transfer radical polymerization mechanism [70]. M = vinyl monomer, M_i_ = polymer with *i* repeat units, X = halide, Mt = transition metal with oxidation state *m*, and L = ligand. Arrows show reaction direction.

As is readily apparent in the above discussion, polymers formed by chain-growth free radical polymerization often exhibit broad molecular weight distributions. These broad dispersities are readily explained by the prevalence of termination reactions that occur when high concentrations of free radicals are present; nevertheless, there is interest in preparing polymers with well-defined structures and molecular weight distributions that approach uniformity (*i.e.*, a *Đ* of 1). Over the past several decades, multiple strategies have been developed for producing polymers with relatively narrow molecular weight distributions via radical-mediated mechanisms. Arguably the best known of these is ATRP [70], where the active radical responsible for propagation is in equilibrium with an inactive form that cannot propagate. By having the equilibrium predominantly lie towards the inactive form, radical concentrations are minimized, greatly decreasing the probability of bimolecular termination reactions. ATRP is initiated when a bromo-containing initiator reacts with a transition metal complex, to yield both a radical capable of propagation and a metal complex with an increased oxidation state; as the equilibrium is strongly shifted away from these products, the radical is only available to participate in propagation reactions for a very short period (see Figure 8). There are several variants of ATRP, with ARGET (activator regenerated by electron transfer) ATRP being particularly relevant for this discussion. ARGET ATRP includes a reducing agent, often ascorbate, that can be used to regenerate the metal catalyst and allows for lower concentrations of metal catalysts to be used [70]. Thus, the complete initiating system for ARGET ATRP comprises a bromo-initiator, a metal complex, and ascorbate.

The work by di Lena and coworkers described in the previous section utilized an initiating system that resembles the ones used in ARGET ATRP, with the transition metal complex being replaced by laccase [69]. Expanding upon their initial work, this group further developed these enzyme-mediated initiating systems and found that a ternary initiating systems comprising a metalloprotease (either laccase, HRP, or catalase), an organobromide initiator, and ascorbic acid would polymerize poly(ethylene glycol) methyl ether acrylate; the resulting polymers had M_n_s of less than 12 kg/mol and *Đ*s of less than 1.7 [71]. While the dispersities achieved here were still higher than those attainable with ATRP, this was still an improvement from the *Đ* of ~1.9 reported in their previous work [69]. Interestingly, similar results were observed for three very different enzymes; while all were metalloenzymes (*i.e.*, enzymes with a metal-containing prosthetic group), each enzyme participates in quite different reactions, with HRP and laccase generating radicals from hydrogen peroxide and oxygen, respectively, while catalase catalyzes the destruction of hydrogen peroxide to oxygen and water. Furthermore, the protein structures are all quite different from each other as both HRP and catalase are iron-containing enzymes (with a heme prosthetic group), while laccase is copper-containing. Despite the differences, the mechanisms of all three enzymes are likely similar for enzyme-mediated ATRP―all of which are distinct from their traditional roles. Here, the metal-center of the enzymes likely reacted with the bromine-containing initiator to yield a radical that could undergo propagation reactions to grow the polymer chain, revert to an inactive form, or undergo *Đ* increasing termination reactions. The exact nature of the redox reaction between the bromo-containing initiator or polymer and the enzyme is unclear; for the heme-containing enzymes, this could have either been Fe(III) reacting with the bromine-containing initiator to form Fe(IV) or it could have been Fe(II), formed by the reaction between ascorbate and Fe(III), reacting with the bromine-containing initiator to form Fe(III). In either case, the oxidized form of the heme prosthetic group can subsequently be regenerated to its reduced form by the reaction with ascorbate in a process that resembles ARGET ATRP.

A similar approach was developed independently by Bruns and coworkers who explored HRP as an ATRP catalyst [72]. Here, a ternary initiating system composed of HRP, ascorbate, and an organobromide initiator was used to initiate the polymerization of *N*-isopropylacrylamide (NIPAAm), resulting is polymers with M_n_ of 55–220 kg/mol and *Đ* as low as 1.44—a higher molecular weight and slightly narrower *Đ* than di Lena, although the *Đ* is still significantly greater than observed in conventional ATRP. In addition to using HRP, Bruns and coworkers utilized hemoglobin as an ATRP catalyst, a novel approach to enzyme-mediated ATRP [73]. Using conditions similar to those in their HRP system, they found that PNIPAAm could be synthesized with a M_n_ of approximately 72 kg/mol and *Đ* of 1.8. As hemoglobin contains thiols that could potential act as chain transfer sites, leading to higher *Đ*, the experiments were repeated with the hemoglobin thiols blocked, yielding polymer with a *Đ* of ~1.5. When used with other monomers, including PEG acrylate or methacrylate, low M_n_ polymers (<7 kg/mol) could be formed with *Đ* < 1.2, indicating good control over dispersity at relatively low molecular weights. In a novel polymerization technique, they also utilized erythrocytes (*i.e.*, red-blood cells) as a catalyst and found that PNIPAAm was formed with M_n_ of 24 kg/mol and *Đ* of approximately 3.5. Hemoglobin in the erythrocytes likely functioned as a catalyst necessary for initiation as well as a chain transfer agent. Indeed, the dispersity observed here likely resulted from the potential for chain transfer reactions with the hemoglobin and the many other molecules present.

While previous examples have shown that HRP and laccase are capable of initiating polymerization reaction reactions through their peroxidase and oxidase functionality, respectively, their roles in enzyme-mediated ATRP are quite different. The same is true for catalase and hemoglobin. Rather than in its function as a (per)oxidase, it is the enzyme’s metal-containing prosthetic group reacting with an organobromide that initiates the ATRP-like polymerization. This raises an obvious question: could the metal-containing prosthetic group, independent from the rest of the enzyme, initiate ATRP? This has been addressed by several groups [74,75,76], including Kadokowa and coworkers who initiated the ATRP of NIPAAm by replacing HRP with hematin [74], an iron-containing porphoryin similar to the HRP prosthetic group. Thus, with a ternary initiating system of hematin, ascorbate, and a bromo-initiator, water-soluble NIPAAm could be polymerized with similar results to those attained with HRP. This concept was further investigated by the Matyjaszewski group who examined the use of similar iron-containing porphoryins [77]. Their most successful material was mesohemin, a modified form of hemin (a porphoryin similar to hematin); polymerized PEG methacrylates were produced with M_n_ greater than 60 kg/mol with a *Đ* of approximately 1.2—much lower than the previous enzyme-mediated ATRP attempts.

While these porphyrin-based enzyme-mimetics demonstrate superior performance over enzymes as ATRP catalysts, Bruns and coworkers offered an example where the size of the enzyme was a distinct and necessary attribute [78]. They encapsulated HRP within a poly(dimethylsiloxane)-block-poly(2-methyl-2-oxazoline) polymersome and induced pores through the polymersome walls via a photo-mediated reaction with a hydroxyalkyl phenone. While the HRP was too large to diffuse out of the porous polymersome, small molecules were able to diffuse inside, allowing the particles to be utilized as nanoreactors where enzyme-mediated reactions could proceed. By adding PEG acrylate as monomer along with the other components of an ATRP initiating system (ascorbate and bromo-initiator), all of which were capable of diffusing into the polymersome, enzyme-mediated ATRP generated polymer trapped within the polymersome. Molecular weights of about 3 kg/mol were achieved with *Đ* of 1.55.

**Figure 9:**
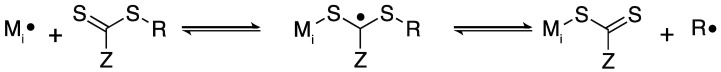
Reaction between a radical on a growing chain and a reversible addition fragmentation chain transfer (RAFT) agent [79]. This is the key process in RAFT polymerization.

In addition to their role in ATRP, enzymes have been utilized in other controlled polymerization reactions, notably RAFT. By adding a RAFT agent, the number of radicals present at any time is reduced and the chances for termination are decreased (see Figure 9); this leads to the generation of polymers with narrower molecular weight distributions [79]. In an early example by di Lena and coworkers that hinted at enzyme-mediated ATRP, the addition of a RAFT-agent to a ternary initiating system of laccase, ascorbate, and a bromo-initiator permitted the generation of polymer with *Đ* ~1.35 from poly(ethylene glycol) methacrylate, much lower than the >1.94 *Đ* attained in the absence of the RAFT agent [69]. In a more thorough examination of enzyme-mediated RAFT, An and coworkers employed the HRP/acetylacetone/hydrogen peroxide ternary combination [80]. They utilized HRP-mediated RAFT to polymerize several monomers, including *N*,*N*-dimethylacrylamide, poly(ethylene glycol) acrylate and *N*-vinyl pyrrolidone, with impressively low dispersities—for *N*,*N*-dimethylacrylamide, polymers with M_n_ between 20 and 60 kg/mol all had *Đ* < 1.1 and a sample with M_n_ of nearly 500 kg/mol had a *Đ* of 1.28. This group also demonstrated that RAFT could be performed under aerobic conditions by utilizing the GOx-HRP bienzymatic system [80]. The initiating system of GOx, HRP, glucose, and acetylacetone was used to both generate radicals in the presence of oxygen and to remove oxygen that could otherwise interfere with propagation. There was some precedent for using GOx in RAFT as an oxygen scavenger as this technique was successfully demonstrated by Stevens and coworkers [81]; while no enzymes were directly involved with initiation or propagation, the presence of GOx permitted RAFT to be performed under aerobic conditions. The An group built on this by use of the bienzymatic GOx-HRP system, where enzymes played multiple roles in the polymerization reaction; the use of GOx again permitted RAFT to be performed under aerobic conditions [80].

### 2.3. Thiol–ene Polymerization

**Figure 10:**
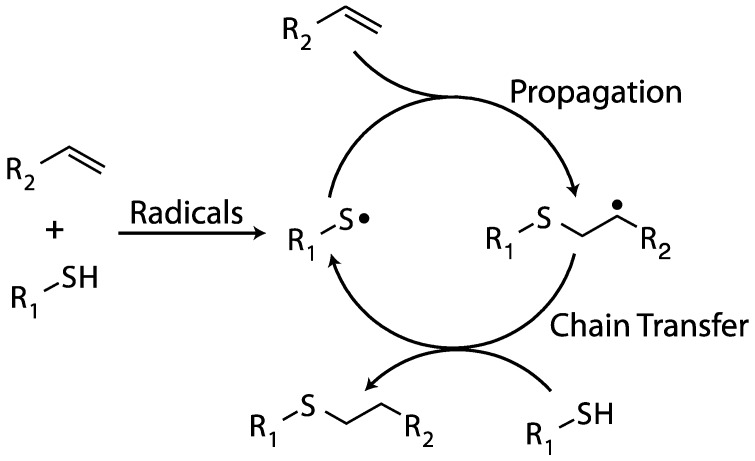
Thiols and electron-rich carbon-carbon double bonds form thioether linkages via radical-mediated, alternating propagation and chain transfer reactions [82,83]. Arrows show reaction direction.

The thiol–ene polymerization, an anti-Markovnikov addition between a thiol and an electron-rich carbon-carbon double bond (e.g., vinyl ether or allyl ether), is a radical-mediated reaction that, unlike the chain-growth vinyl polymerization described above, proceeds through a step-growth mechanism [82,83]. Initiation of thiol–ene polymerization commences upon the generation of radicals which can either add across a carbon-carbon double bond, affording a carbon-centered radical, or abstract a hydrogen from a thiol monomer, yielding a sulfur-centered thiyl radical. Subsequently, the reaction proceeds via alternating propagation and chain transfer events where a thiyl radical initially adds across a double bond to afford a carbon-centered radical. Owing to the use of electron-rich carbon-carbon double bonds, this carbon-centered radical is unable to propagate; however, it is able to participate in a chain transfer reaction with the ubiquitous thiol, abstracting a thiol hydrogen and yielding the final product, a thioether linkage, and regenerating a thiyl radical (see Figure 10). As this is a step-growth mechanism, the thiol and carbon-carbon double bonds are consumed in a 1:1 stoichiometric ratio. Whereas monofunctional reactants will not polymerize, only coupling together, difunctional reactants will form linear polymers and multi-functional monomers are necessary to form cross-linked polymer networks. A distinguishing characteristic of the thiol–ene polymerizations is that, unlike radical-mediated vinyl polymerizations, they are extraordinarily resistant to oxygen inhibition; the peroxy radical generated by the addition of the carbon-centered radical to oxygen is able to abstract a thiol hydrogen, permitting the cycle of alternating propagation and chain transfer reactions to continue. This inherent tolerance to oxygen permits thiol–ene polymerization to proceed under atmospheric conditions and suggests that it is well-suited for oxygen-mediated initiation.

In work performed by two authors of this review [84], glucose oxidase, in combination with glucose and Fe^2+^ salts, was used to polymerize aqueous thiol–ene monomer formulations by exposing them to oxygen. Analogous to the acrylate systems investigated by both Iwata [41] and Bowman [42], described above, GOx was used to oxidize and reduce glucose and oxygen to gluconolactone and hydrogen peroxide, respectively; the hydrogen peroxide was then converted to hydroxyl radicals through the Fenton reaction involving Fe^2+^. An unfortunate limitation of this initiating system was the difficulty associated with increasing reaction rates, owing to several potential inhibitory and terminating reactions capable of consuming radicals, reaction rates decrease beyond an optimal Fe^2+^ concentration (approximately 7.2 × 10^−5^ M). Indeed, the role of Fe^2+^ and Fe^3+^, generated from the Fenton reaction, is highly complex with many reactions either generating or eliminating radicals [84]. Complicating matters further, Fe^2+^ ions themselves in the absence of GOx are able to slowly generate radicals when exposed to oxygen; two equivalents of Fe^2+^ are used to reduce oxygen to hydrogen peroxide and one additional equivalent is utilized in the Fenton reaction. As the potential for other strategies to increase reaction rates are somewhat limited (glucose concentration certainly influences rates, but increasing its concentration will only increase reaction rates until the saturation point is reached—approximately 0.028 M glucose for 14.8 kU/L GOx), an alternative approach for generating radicals from hydrogen peroxide is needed to circumvent the inhibitory effects of iron salts. Drawing upon the much of the research discussed above, we found that HRP readily initiated thiol–ene polymerization with hydrogen peroxide either exogenously added or generated *in situ* by the action of GOx on glucose and oxygen [84]. Moreover, for the GOx/glucose/oxygen/HRP initiating system, reactions rates steadily increased with HRP concentration (ranging from 26.1 to 261 kU/L), thus overcoming the Fenton chemistry limitations and providing yet another example of a bienzymatic initiating system. Interestingly, in addition to its role as a peroxidase, HRP was capable of promiscuously functioning as a thiol oxidase where it generates thiyl radicals by utilizing molecular oxygen as an oxidant, thus allowing polymerization in the complete absence of hydrogen peroxide [85,86]. Also of note, an external mediator was not required as there was no significant change in reaction rates in the absence or presence of acetylacetone, thus it appeared that one of the other formulation components, almost certainly the thiol, reacted directly with the enzyme to generate initiating radicals.

### 2.4. Oxidative Coupling

**Figure 11:**
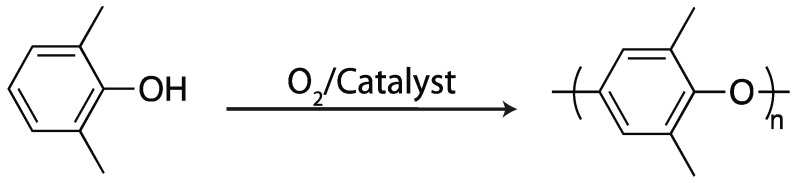
The polymerization of 2,6-dimethylphenol into a poly(phenylene oxide) via oxidative coupling [87].

Phenol and related compounds will polymerize via an oxidative coupling mechanism in the presence of oxygen and a suitable catalyst (often copper salts and amines) (see Figure 11). This process is best known for the commercial manufacture of poly(phenylene oxide) (PPO) from 2,6-substituted phenols via a polycondensation reaction, generating water as a byproduct, with a complex reaction mechanism that exhibits characteristics of both chain- and step-growth polymerization [87]. While the details of the reaction mechanisms are quite different from both vinyl chain-growth and thiol–ene step-growth polymerization, the utilization of oxidases and peroxidases to effect polymerization via oxidative coupling shares many characteristics with the other enzyme-mediated polymerization mechanisms.

#### 2.4.1. Horseradish Peroxidase

In many early examples, HRP was used to remove phenols from water by initiating oxidative coupling reactions that yielded dimeric, oligomeric and polymeric products [25,26]. The intention here was not to produce polymers for use, but rather to aid in the removal of toxic pollutants; these products tend to be insoluble and readily precipitate, allowing for their facile removal by filtration. To illustrate, HRP and hydrogen peroxide were added to aqueous solutions of phenols to generate the readily separable higher molecular weight products [25]. In contrast to the behavior observed with vinyl polymerization, phenol reacted directly with HRP and thus no additional mediator (e.g., acetylacetone) was required. Other than this difference, the utilization of HRP for either vinyl polymerization or oxidative coupling has many similarities, including a tendency for HRP to be deactivated by excess hydrogen peroxide; one strategy to inhibit HRP-deactivation was to add poly(ethylene glycol) as it seemed to offer some protection to the enzyme and extended its useful lifetime [25]. Beyond this early work, the pollution removal capabilities of HRP continue to be developed. Tang and coworkers combined the HRP/hydrogen peroxide system with a composite made of graphene oxide and nano-Fe_3_O_4_ (GO/Fe_3_O_4_) and explored the capabilities of these materials for removing 2,4,-dichlorophenol (DCP) [27]. The HRP/hydrogen peroxide system was shown to remove 35% DCP within 2 h, while the GO/Fe_3_O_4_ composite on its own would only remove 9%. Combining the two systems demonstrated a synergistic effect where 93% of the DCP was removed, most of which occurred in the first 30 min. Other research has explored methods for preventing the deactivation of HRP by hydrogen peroxide. In efforts by Lopez-Gallego and coworkers [88], hydrogen peroxide was generated *in situ* by the action of two enzymes. This trienzymatic system, reminiscent of the GOx/HRP bienzymatic cascade discussed previously, used formate dehydrogenase, nicotinamide adenine dinucleotide (NADH) oxidase, and HRP, along with two redox cofactors, oxidized nicotinamide adenine dinucleotide (NAD^+^) and flavin mononucleotide (FMN). First, by the action of formate dehydrogenase, formic acid was oxidized to carbon dioxide while NAD^+^ was converted to its reduced form (NADH). The reduced NADH underwent a redox reaction with FMN, regenerating the oxidized NAD^+^ concomitantly with the reduced FMNH_2_ which, by the action of NADH-oxidase, was oxidized back to FMN as molecular oxygen was reduced to hydrogen peroxide. Finally, by the familiar behavior of HRP, phenolic contaminants were polymerized. Interestingly, this enzymatic cascade did not function when all the enzymes were solubilized; while this inhibitory mechanism was not elucidated, it appears likely that one of the enzymes may have been deactivated by the presence of hydrogen peroxide. Nevertheless, when the formate dehydrogenase and NADH-oxidase were immobilized on glyoxal-functional agarose beads and HRP on boronate-functional agarose beads, this trienzyme system functioned well and proved capable of removing phenolic contaminants.

In addition to its role in phenolic contaminant removal, HRP has also been explored as a means to generate useful polymeric materials. As many phenolic compounds, as well as their potential polymeric forms, have limited solubility in water, these reactions are often performed in mixed solutions of water and organic solvents. In an early example by Dordick and coworkers [89], the polymerization of 4-phenylphenol and other substituted phenols in water/dioxane solutions was demonstrated. While the activity of HRP decreased as the dioxane concentration was increased, it still remained quite active at dioxane concentrations as high as 80% or 90%. This is important as substituted phenols have low solubility in water and their oligomeric derivatives even less so, thus the production of higher MW products necessitated the utilization of solvent blends with significant dioxane concentrations. For example, a solution of 4-phenylphenol in 10% dioxane and water, with the addition of HRP and hydrogen peroxide, formed oligomeric products of only 0.5 kg/mol, whereas increasing the concentrations of dioxane to 85% led to products with M_n_ of 26 kg/mol. This effect was largely attributable to the increased solubility of the products in the water/dioxane solvent blends.

In a demonstration of how enzymatic polymerization leads to the facile synthesis of functional macromolecules, Cui and coworkers polymerized pyrogallic acid to generate moderately-high molecular weight polymers [90]. Adding HRP and hydrogen peroxide to pyrogallic acid solutions in water/solvent blends, again necessary to solubilize both the monomers and generated polymer, resulted in poly(pyrogallic acid) polymers with M_n_ as high as 39 kg/mol; these materials were found to have excellent antioxidant properties, exceeding those of commonly-used commercial materials.

Difficulties with solubility can be avoided by utilizing water-soluble, phenol-functionalized materials. This approach has been utilized to produce cross-linked hydrogels, where phenol-functionalized polysaccharides have been regularly polymerized by the HRP/hydrogen peroxide initiating system. To give two examples by different groups, both phenol-functional alginate, examined by Sakai and coworkers [91], and dextran, explored by Feijen and coworkers [92], rapidly polymerize after exposure to HRP and hydrogen peroxide, with both systems achieving gel times of less than 10 s. Both groups also noted that gelation times increased at high hydrogen peroxide concentrations, attributable to HRP deactivation. As mentioned previously, a bienzymatic system where GOx generates hydrogen peroxide *in situ* can be utilized to circumvent the HRP deactivation, an approach that has been explored at length [93,94,95,96,97,98,99].

Lignin, a complex aromatic macromolecule found in cell walls that is present as a waste product in paper pulping processing, is currently under-utilized as a natural resource. By having facile methods available for modifying lignin, this waste material could be readily incorporated into polymeric industrial products (e.g., paint, coatings, and adhesives). It has been found that sulfonated lignin can be utilized as an industrial dispersant, with tremendous improvement in properties observed at higher molecular weights. Qiu and coworkers were able to increase the molecular weight of sulfonated lignin six-fold via polymerization initiated by HRP and hydrogen peroxide [100].

In addition to phenolic monomers, other monomers have been polymerized by HRP-mediated oxidative reactions, including those used to make conductive polymers [101,102]. Samuelson and coworkers utilized the HRP/hydrogen peroxide initiating systems to generate polyaniline (PANI) [101]. A major challenge in the enzyme-mediated polymerization of aniline is its lack of water-solubility. While there have been many reports on methods to circumvent its poor water solubility (e.g., using solvent blends, or by emulsion polymerization), the resulting product is typically not the desired benzenoid-quinoid form of the polymer, instead forming a branched structure that limits conjugation length and the resulting conductivity. The approach by Samuelson involved polymerizing aniline in the presence of a polyelectrolyte template, sulfonated polystyrene, that both aided polymer solubility and promoted monomer alignment. The resulting PANI demonstrated conductivity that was readily controlled by changing process conditions. Similarly, this approach of using sulfonated polystyrene as template was also utilized in the formation of another conductive polymer, poly(3,4-ethylenedioxythiophene) (PEDOT), where the monomer, 3,4-ethylenedioxythiophene, was polymerized in the presence of the template and HRP/hydrogen peroxide as the initiating system [102].

#### 2.4.2. Glucose Oxidase

**Figure 12:**
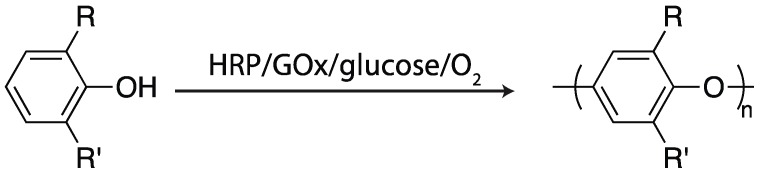
Utilizing horseradish peroxidase (HRP), glucose oxidase (GOx), glucose, and oxygen as a quaternary initiating system for polymerizing substituted phenols [96].

For HRP-mediated polymerization reactions performed in a reactor and intended for subsequent utilization, hydrogen peroxide can be added at any arbitrary rate, minimizing the concentration at any time, and mitigating deactivation of the enzyme. This approach, however, is not possible for any type of *in situ* polymerization. Thus, it is useful to be able to generate the necessary hydrogen peroxide only as it is needed; this approach has been employed, as has been already seen in several examples discussed above, for vinyl and thiol–ene reactions, where GOx generates hydrogen peroxide *in situ*, which can then be used by further reaction with a peroxidase to initiate polymerization [95,96,97,98,99]. The apparent earliest utilization of this bienzymatic system, by Kobayahsi and coworkers, was for the polymerization of substituted phenols [96]. Phenol and several 4-alkylphenols, which the alkyl groups ranging from methyl to pentyl, were polymerized using the GOx/glucose/oxygen/HRP quaternary initiating system (with both enzymes at a concentration of 2 g/L) with no external mediator required (see Figure 12). The polymers generated had M_n_ as high as 13 kg/mol, with the solubility of the polymer likely the limiting factor for molecular weight. This method was readily adapted for the production of hydrogels by using water-soluble, phenol-functionalized polymers, including hyperbranched polyglycerols [95], alginates [98], and poly(vinyl alcohol) [99]. As this technique has been utilized with biocompatible polymers, it holds promise for the development of scaffolds for living cells and biomedical adhesives, suitable for hemostats and wound closures, as has been demonstrated on rat models [99].

#### 2.4.3. Laccase

As was the case for vinyl polymerization, laccase has also been utilized for initiating polymerization by oxidative coupling. The reaction proceeds readily in the presence of molecular oxygen and phenolic compounds, without requiring an additional mediator. The laccase-mediated oxidative coupling of phenols bears a strong resemblance to the HRP-mediated reaction—many of the applications are quite similar—though, since laccase utilizes oxygen as an oxidant, one crucial difference is the lack of enzyme deactivation upon hydrogen peroxide exposure. In a fairly simple example, laccase has been investigated as a method for generating polymers from phenolic compounds and related materials [103]. The molecular weights of polymers made from phenol or 4-*tert*-butyl phenol were typically in the 1–2 kg/mol range. Other monomers resulted in polymers of higher molecular weight, with polymers made from *m*-cresol having M_n_ approximately 15 kg/mol and those from bisphenol A over 21 kg/mol. As the reactions were performed in aqueous solutions of solvents, molecular weight was likely influenced by the solubility of the generated polymer.

Laccase-mediated reactions were also investigated as a means to generate small molecule products from phenolic precursors [104]. This reaction was performed in ethanol/water solutions and, unlike with vinyl polymerizations, no external mediator (e.g., acetylacetone) was required. While some small molecule products were produced, there was also significant amounts of undesirable oligo- and polymeric products formed. In utilization similar to that of HRP, this ability to generate insoluble products from phenolic compounds has been utilized for the removal of contaminants from waste streams, including bisphenol A [66,105,106] or 1-naphthol [61]. There is particular interest in developing methods for the facile removal of bisphenol A, as this compound is used in the production of many commercial polymeric products and has numerous deleterious medical attributes associated with it.

In an application similar to an HRP-mediated one described above, laccase has been utilized to modifying lignin, a phenolic waste product obtained from paper mills [60]. In efforts to find facile methods for making use of this common waste product, a laccase-mediated reaction was utilized to increase the molecular weight of lignin six-fold; their were also extensive modifications to the functional groups present, with carbonyl and aliphatic hydroxyl groups becoming more prevalent as the concentration of methoxyl and aromatic hydroxyl groups decreasing. As noted previously [100], increasing the molecular weight of lignin tends to improve its performance as an industrial dispersant and it seems likely that both laccase and HRP are capable of modifying lignin for this purpose.

As is the case for HRP, laccase is also capable of polymerizing non-phenolic monomers via oxidative coupling, often for the production of conductive polymers [63,107]. As the monomers, including pyrrole [63] and 4-aminodiphenylamine [107], and resultant polymers are sparingly water soluble, alternative methods are required to prevent precursors and products from precipitating. Thus, enzymatic polymerization reactions using these monomers have been performed in the presence of vesicles formed by sodium bis-(2-ethylhexyl)sulfosuccinate, a common industrial surfactant widely known as AOT. While the AOT vesicles assisted in keeping the generated polymer dispersed they also acted as templates for polymerization, decreasing the number of defects in the polymer and helping improve the electrical properties by increasing conjugation length [63,107]. This vesicle template utilization was similar to that of the sulfonated polystyrene discussed above in HRP-mediated reactions [101,102].

## 3. Formulation Considerations for Enzymatic Polymerizations

### 3.1. Enzyme Deactivation

When performing HRP-mediated polymerization, it is especially important to consider both the beneficial and deleterious roles hydrogen peroxide plays. Hydrogen peroxide is both responsible for initiating polymerization, where is serves as the oxidant, as well as inhibiting it by deactivating HRP. As HRP deactivation tends to occur at high hydrogen peroxide concentrations, it can be mitigated by maintaining a low hydrogen peroxide concentration throughout the reaction. This is readily accomplished either by adding hydrogen peroxide slowly to a reaction flask, or by generating hydrogen peroxide *in situ* by the action of, for example, GOx.

Other approaches rely on somehow protecting the enzyme, such as the addition of PEG that has been shown to increase enzyme stability, although the additional protection afforded at high PEG concentrations has been shown to be limited [25]. In a somewhat more sophisticated protection strategy, enzymes can be immobilized on a solid support. As an illustration, HRP was immobilized on silicon wafers, permitting facile removal and subsequent reuse [44]. This can be particularly important as the ability to reuse enzymes improves their economic viability in industrial processes. In another example, cross-linked aggregates of versatile peroxidase, an enzyme with HRP-like characteristics, and GOx, formed through aggregation and subsequent cross-linking via glutaraldehyde, functioned as a self-immobilized system [97]. Enzymes can also be immobilized within a hydrogel, such as the example where a GOx/HRP bienzymatic system was utilized to generate a nanocomposite hydrogel from water-soluble methacrylates and CNO nanosheets [54], to improve the stability of the incorporated enzymes.

Of course, these strategies to prevent deactivation may be combined. One approach, used for the removal of phenolic contaminants, utilized an immobilized trienzymatic system (formate dehydrogenase, NADH-oxidase, and HRP) capable of polymerizing phenolic compounds in the presence of oxygen and formic acid [88]. For this example, by immobilizing the enzymes onto agarose beads, the enzymatic stability was greatly increased. Interestingly, results were poor when all three enzymes were immobilized on the same support, whereas the efficiency was greatly increased when the HRP was immobilized on separate agarose beads.

### 3.2. Enzyme Promiscuity

While high specificity is often cited as an advantage of enzyme-mediated reactions, there are many cases were enzymes behave in a promiscuous manner by catalyzing reactions for multiple substrates [108]. In examples relevant to polymerization, both GOx and HRP have been observed to catalyze multiple reactions. HRP, which as a peroxidase normally makes use of hydrogen peroxide as an oxidant, can also utilize oxygen as oxidant and has been shown in several studies to act as a thiol oxidase [84,85]. In work performed by two of the authors of this review, where a GOx/HRP bienzymatic system was utilized to perform oxygen-mediated thiol–ene polymerization, it was found that not only was the addition of a mediator not required as the thiol groups present on the monomers appeared capable of acting as substrates, but hydrogen peroxide was not required either [84]. Instead, HRP catalyzed the concurrent reduction of oxygen and oxidation of thiols, likely forming polymerization-initiating thiyl radicals. Luckily, this unexpected alternative reaction path was not problematic as it was used in a system where oxygen was intended to initiate the reaction. Indeed, such a lack of specificity may in some cases be advantageous as it permits non-canonical reactions to utilize the benefits of enzyme-mediated reactions. For example, GOx was been shown to catalyze reactions in the absence of oxygen by instead interacting with *n*-hydroxyimide moieties present on a modified heparin that, in the presence of glucose, generated radicals to initiate the polymerization of acrylamide monomers [56].

Nevertheless, polymerizations effected by an unexpected substrate could be detrimental, especially for applications that rely upon *in situ* polymerization as it could either lead to premature polymerization in an adhesive formulation or a false positive for signal detection. In work by Stevens and coworkers that utilized a GOx/HRP bienzymatic system for polymerizing acrylamide monomers, they found that acetylacetone, the mediator required by HRP, could be directly oxidized by GOx [55]. While they readily accounted for this reaction in their sensing application by careful calibration, it serves as a cautionary tale and reminds us that any potentially unwanted reactions need to be thoroughly investigated and accounted for.

### 3.3. The Role of Mediators

As discussed above, HRP-mediated vinyl polymerization usually requires the presence of a suitable mediator, often a β-diketone and usually acetylacetone [22]. While mediators are not absolutely necessary for initiating polymerization, their absence requires excessive amounts of hydrogen peroxide which will almost certainly lead to HRP deactivation. The presence of a mediator permits drastically lower concentrations of hydrogen peroxide to be used, decreasing the rate of HRP deactivation and permitting the utilization of hydrogen peroxide generated *in situ* by GOx. While the presence of a mediator generally leads to no deleterious effects, its presence does need to be thoroughly investigated to ensure that it does not participate in any unexpected reactions (as was mentioned above in the enzyme promiscuity discussion). Although acetylacetone is the most common mediator used for enzyme-mediated vinyl polymerizations, other meditators have been investigated. As the mediator is incorporated into the polymer chain, opportunities exist for either post-polymerization functionalization of the chain ends or grafting polymers onto other molecules. As an example of the latter, triblock copolymers of PEG-PPG-PEG were utilized as mediators for laccase-mediated polymerization where hydrogels were formed from aqueous solutions of PEGDA [67]; the PEGDA network that formed was grafted onto the PEG-PPG-PEG triblock copolymers. The motivation for using a macromer mediator was that the generated hydrogel would have both hydrophilic and hydrophobic regions, a desirable attribute for a material intended for use in environmental remediation as it would allow the enzyme to remove both hydrophilic and hydrophobic contaminants.

Not all enzyme-mediated polymerization chemistries require mediators. For enzyme-mediated thiol–ene chemistry, the thiol monomer was able to serve as substrate—the inclusion of acetylacetone had negligible influence on the reaction kinetics [84]. Similarly, the phenolic monomers utilized in oxidative coupling reactions can also serve as enzyme substrates, obviating the need for an additional mediator. In addition to the many occurrences where phenols function as both mediator and monomer, phenols are also capable of functioning as mediators in other polymerization reactions. For example, solutions of thiol-functionalized multi-arm PEG, HRP, and phenolic compounds form gels in aerobic conditions [93]. The mechanism is seemingly quite complex and may not be fully-elucidated; it was hypothesized by Kamiya and coworkers that non-enzymatic, *in situ* generated hydrogen peroxide, produced via thiol oxidation, reacted with the phenolic compounds to yield radicals capable of abstracting thiol hydrogens [93]. The resulting sulfur-centered thiyl radicals could combine, leading to disulfide formation. Given that it has also been established that thiols are suitable substrates of HRP, the role of the phenols in this work is unclear, although it appears their presence did have some positive influence on reaction rates.

## 4. Applications Utilizing Enzyme-Mediated *in Situ* Polymerization

### 4.1. Adhesives/Biomaterials

As enzymes can be stable in an aqueous, pH-controlled milieu, enzyme-mediated polymerization is particularly well-suited for the fabrication of hydrogels, where the initiating action of the enzyme effects the transition of free-flowing viscous solutions to cross-linked polymeric networks. These hydrogels may have sufficient mechanical strength to function as adhesives. Moreover, as aqueous-based materials can be formulated with biocompatible materials, such hydrogel adhesives may be useful for biomedical and surgical applications. Indeed, while there are many adhesives currently utilized for medical procedures, including alkyl cyanoacrylates and fibrin-based materials, these materials each have significant disadvantages. For example, although cyanoacrylates display good physicomechanical properties, there are serious concerns over the toxicity of their hydrolysis products [109,110,111,112]. Conversely, fibrin-based adhesives are generally thought of as safe, but are limited by their poor mechanical strength [113,114,115]. Given these limitations, there is a clear need to develop new classes of biomedical adhesives and enzyme-mediated polymerization appears well-suited to aid in this goal.

There are several methods commonly utilized to trigger liquid-to-gel transformations of adhesive materials. For the so-called two-part adhesives, polymerization commences upon mixing of two components; common examples include epoxy adhesives, which consist of separately-stored epoxy and amine formulations, and the aforementioned fibrin sealants, consisting of fibrinogen and thrombin preparations. An inherent disadvantage of this method is the potential for inaccurate mixing of the two components, leading to less-than-desired physical properties and potential adhesive or cohesive failure. Other strategies, including the humidity-mediated polymerization of alkyl cyanoacrylates, rely upon an environmentally-borne initiation stimulus to effect gelation. For one of the more fully-developed enzyme-mediated examples, a hydrogel wound dressing was formulated from a phenol functional poly(vinyl alcohol) and the GOx/HRP combination [99]. In order to effect polymerization, this system requires both GOx substrates glucose and oxygen. By omitting the glucose, this formulation would only solidify once it is in contact with a glucose source—in this case, blood. Thus, the hydrogel formation only proceeds once the formulation is applied to a bleeding wound. In experiments that used glucose solution in bovine serum albumin as a blood substitute, gelation times of less than 10 s were readily attainable. Encouragingly, when utilized as wound dressings on rats, the hydrogels not only seemed to aid in wound healing but also exhibited no signs of bio-incompatibility from either of the residual enzymes.

An alternative approach would be to include glucose in the formulation and utilize molecular oxygen, ubiquitous in the atmosphere, as the environmentally-borne stimulus. This approach has been demonstrated for GOx/HRP-mediated thiol–ene polymerization [84]. A formulation of thiol and ene monomers with glucose, GOx and HRP could be applied via spray, where the readily accessible surfaces of atomized droplets would permit rapid oxygen diffusion, leading to polymerization initiation. An advantage here, over the formulations that rely on blood glucose, is the decreased risk in unpolymerized material remaining. In addition to the GOx/HRP pair, there are likely other enzymatic methods of inducing oxygen-mediated *in situ* polymerization, notably laccase as it is capable of forming radicals directly from molecular oxygen.

### 4.2. Sensors

Enzymes have often been incorporated in devices for the purpose of molecular sensing, where an analyte of interest, functioning as an enzyme’s substrate, generates a readily detectable compound. An especially well-known example is the use of GOx for glucose detection where either hydrogen peroxide production or oxygen consumption can be monitored and correlated to glucose concentration. As has been noted extensively, GOx is also capable of catalyzing the formation of hydrogels; doing so results in the GOx being embedded within the gel [116]. The now-immobilized GOx retains most of its activity, permitting the hydrogels to be used as glucose sensors—this is useful as enzyme immobilization affords greatly increased stability. Of course, the enzyme responsible for polymerization does not need to be the enzyme utilized for sensing. For example, a sensor capable of detecting reactive oxygen species via incorporated catalase and superoxide dismutase, enzymes that generate molecular oxygen from hydrogen peroxide and superoxide, respectively, was produced by the laccase-mediated polymerization of acrylate-functionalized chitosan [68]; in this system, the generation of oxygen bubbles permitted facile detection via ultrasound imaging. The utilization of laccase here was necessitated by the presence of catalase, an enzyme that rapidly converts hydrogen peroxide to water and oxygen; this swift hydrogen peroxide removal precluded the use of peroxidases like HRP.

While these examples only utilize the enzyme-mediated polymerization reaction as a means to produce the enzyme-containing scaffold, the polymerization process itself can also participate in the sensing mechanism. Because polymerization generates a large effect (the generation of polymers from many propagation reactions) from a single event (the generation of a radical), polymerization can be used to amplify a signal, allowing for the detection of analytes even at very low concentrations. For example, Bowman and coworkers devised a system where a biotin-tagged protein could be bound to a GOx-avidin conjugate; the bound GOx could then, in the presence of a formulation with Fe^2+^ salts and glucose, initiate the polymerization of acrylate monomers [51]. By including a fluorescent acrylate monomer in the polymerizable formulation, the resulting polymer was also fluorescent and readily detected by a plate reader. An advantage of this technique was that, owing to the previously-demonstrated oxygen tolerance of GOx-mediated polymerization, it permitted analyte detection under aerobic conditions. The use of GOx-mediated detection was further developed by Stevens and coworkers [55], where the familiar GOx/HRP pair was used to initiate the polymerization of 3-aminopropyl methacrylamide (APMA) in the presence of gold nanoparticles; as mentioned above, the formation of polymer was sufficient to induce aggregation of gold nanoparticles, leading to a color change. As the polymerization required that all of the components of the initiating system be present, this method could be utilized for detecting the presence of a missing initiating system component. For example, by omitting HRP, this system was utilized as a means of detecting the presence of HRP at concentrations as low as 250 ng/mL. Moreover, by including HRP and using a reverse assay method, this could also be used to detect catalase; as the GOx-generated hydrogen peroxide was required by HRP to initiate polymerization, the presence of a catalase, a hydrogen peroxide destroying enzyme, prevented polymerization as well as the subsequent aggregation-induced color change. With this technique, detection of catalase down to 0.7 ng/mL was attainable.

## 5. Conclusions

Oxidoreductases, including HRP, GOx, and laccase, have been deployed, in variety of roles (e.g., initiator, catalyst, oxygen scavenger), to perform a broad range of radical-mediated polymerization reactions, including the chai*n-*growth polymerization of vinyl monomers, the step-growth polymerization of thiol–ene formulations, and the radical-mediated oxidative coupling of phenolic monomers. Moreover, enzyme-mediated reactions have been used to generate polymers in an extraordinarily wide range of environments, from *in vitro* solution and emulsion polymerization, to the *in situ* generation of cross-linked hydrogels, to the formation of polymers on a surface or inside hollow particles. This versatility permits the utilization of enzyme-mediated polymerization in numerous fields, allowing for significant advancements to be made. For example, their biocompatibility and catalytic efficiency under mild reaction conditions make them particularly well-suited for medical applications that utilize *in situ* polymerization, notably for cross-linking of biomedical adhesives or as an amplification mechanism for sensing applications, and their environmental-friendliness makes them particularly advantageous for environmental remediation applications. It is this combination of attributes, as well as the decrease in cost driven by biotechnology advances, that will likely allow enzyme-mediated polymerization reactions to be utilized in an increasing number of fields in the future.
